# What is there to learn about violence and masculinity from a genderqueer man?

**DOI:** 10.1080/16549716.2018.1458937

**Published:** 2018-04-25

**Authors:** Rebecca Helman, Kopano Ratele

**Affiliations:** a Institute for Social and Health Sciences, University of South Africa, Pretoria, South Africa; b Violence, Injury and Peace Research Unit, South African Medical Research Council–University of South Africa, Tygerberg, South Africa

**Keywords:** Violence, masculinity, men, genderqueer, LGBTIQ+

## Abstract

**Background**: In light of the global health burden of violence, which is predominantly perpetrated by men, studies have explored the relationship between masculinities and violence. However, there is a relative lack of work focusing on non-hegemonic men and masculinities in relation to violence. Such work has the potential to advance violence prevention work.

**Objective**: This article aims to show the shifting relationship between constructions of violent and non-violent masculinity in the talk of a genderqueer man. The article also aims to demonstrate how qualitative approaches are able to reveal the complexity and contradiction in accounts of masculine identities as these are negotiated within the context of the research interview.

**Methods**: The article is based on a case study of Adam, a middle-class, ‘white’, ‘genderqueer’ man who participated in a larger study which explored the ways in which gender is constructed within 18 South African families. Adam’s interview is analysed using a Foucauldian discourse analysis.

**Results**: The analysis demonstrates the complex and contradictory process involved in negotiating and resisting a violent version of masculinity. Constructing male violence as rooted in particular psychosocial and cultural assumptions, rather than as an automatic biological response, enables Adam to resist this violence. This deconstruction of violent masculinity is linked to Adam’s ‘genderqueer’ identity or ‘in-betweenness’, which facilitates a critical consciousness in relation to notions of gender. The analysis also demonstrates how notions of masculinity are deliberated and co-constructed within the relational context of the interview.

**Conclusions**: This article shows that resisting and reformulating masculinity in non-violent ways is a complex process. This suggests that violence prevention efforts need to focus on the creation of spaces for ongoing dialogues about non-violence. As demonstrated by the context of the interview, relational, conversational spaces have the potential to facilitate the co-construction of non-violent masculinities.

## Background

Violence is globally recognised as a significant health burden [1]. Across diverse contexts, violence is disproportionately perpetrated by male-bodied persons [2–4]. The notion of ‘masculinity’ (or ‘masculinities’) has emerged as a central way of understanding the relationship between men and violence [5]. Masculinities operate as symbolic resources which inform the self, ‘enabling different forms of masculine and feminine subjectivity, which in turn allow for different possibilities of meaningful action. One such meaningful action is violence’ [6, p. 141]. South Africa provides a particularly instructive example for studying the relationship between violence and masculinity owing to the colonial and postcolonial history of masculinity in the country [3,7] as well as alarmingly high rates of men’s violence [4,8–10].

Although studies have focused on how dominant beliefs about masculinity (and femininity) are associated with high rates of violence [4,8,10–12], they have predominantly focused on cis-gendered (persons whose social gender matches their biological sex) men and women, neglecting other gender identities. Research on LGBTIQ+ people has tended to focus on violence and other forms of victimisation of this group [13–17]. There is an increasing body of work on gender-non-conforming people; however, much of this work focuses on transgender people who identify with, behave or appear like a sex other than that which they were assigned at birth [18]. There is also some research which suggests that those who identify as genderqueer may be at higher risk for violence than people who identify (and pass) as transgender [19]. (The term genderqueer has been used to refer to individuals who see their gender as fluid or hybrid, or reject the binary between male and female [19].) This may be attributed to the perception that non-conventional expressions of gender, for example those which challenge a dichotomous order of gender, are regarded as particularly threatening to social relations [18,19]. However, the association between the risk of violence victimisation and gender identities is very complex and it is necessary to be cautious not to naturalise the threat potential to some identities over others.

As argued in this article, non-conventional gender identities have the potential to destabilise particular versions of masculinity, including violent masculinities. The promotion of gender equality or more egalitarian masculinities has been recognised as central to disrupting practices of violence [20,21]. Such disruption involves challenging and transforming dominating, aggressive and violent versions of masculinity. Yet caution ought to be exercised when considering the complex link between non-conventional gender identities and the potential to destabilise violent masculinities, as this may ascribe a natural ‘transgressive’ status to certain identities. Our use of a case study is intended as an example of some of the possible links and contradictions between issues of gender and violence, rather than as a blueprint which constructs fixed relationships between these issues.

In order to explore the interrelated issues of masculinity and violence, this article presents an analysis of a case study of Adam (pseudonym), who self-identifies as ‘genderqueer’, and uses the pronouns ‘he’ and ‘him’. (Adam’s use of the masculine pronoun was not probed.) He lives in a middle-class suburb in Cape Town. Adam self-identified as ‘white’ during his interview. (Note that the terms ‘white’, ‘black’ and ‘coloured’ were racial categories constructed and widely used under the system of apartheid in South Africa to classify people according to their ‘race’. While we acknowledge the problematic nature of these terms, we also feel it is necessary to acknowledge the reality of racialisation in South Africa – where constructions of race continue to hold social salience.) Adam lives with his wife, his two daughters, of whom the younger is adopted, and another married couple with whom he and his wife are in a polyamorous relationship. While the term ‘genderqueer’ has diverse meanings, in this article it is used to refer to a disidentification with and rejection of the binary categories of ‘male’ and ‘female’. As Adam says, ‘I identify as genderqueer because that’s how far I feel from the sort of defined male roles’. He describes how identifying in this way makes him ‘able and allowed to be more sort of accessible for hugs and cuddles and warm motherly presence’. This is supported by his wife, Hannah, who describes him as ‘much gentler, much more emotional […] much softer’. Adam explains how he and Hannah divide up childcare: ‘We decided together if she was going to do all the breast feeding then I could do all the nappies. So I’ve heard incredulity from a lot of mothers [laughs] and I think I get upset because I see the role of a man in the house to be more equal and I get upset when I get praised for doing what I see as my job as a parent’. This discussion demonstrates how Adam’s (queer) construction of gender is as ‘at odds with the normal, the legitimate, the dominant’ [22, p. 62]. This is highlighted in Adam’s reference to the ‘incredulity’ he receives from others in response to his enactment of an alternative gender. In South Africa, where most men spend 13 times less time than women taking care of children [23], Adam is an unusual man. However, Adam’s non-conformity extends beyond South Africa, as heterosexual men’s unequal participation in childcare is a global concern. Adam’s queer construction of gender also occurs within a broader national context which continues to be marked by heterosexism, transphobia and gender inequality, including dominant constructions of masculinity which scaffold high rates of violence by men against women, men and people of other genders [4].

However, it must be noted that even though Adam problematises the binary between male and female, he continues to position himself in relation to the category of male. For example, Adam notes: ‘There’s enormous shock that I knit, you know as a man’. In light of this contradiction, we have proposed the term ‘genderqueer man’ to highlight the ways in which Adam both challenges and reproduces notions of masculinity and maleness. The use of this term is an attempt to develop language beyond the ‘limitations and pejorative connotations of current social gender-discourse’ [18, p. 94], while simultaneously acknowledging the power of such gender discourse to shape an individual’s experiences.

Adam’s contradictory position as a genderqueer man creates interesting possibilities for exploring how masculinities are resisted and reformulated in non-violent and egalitarian ways. As Foucault has argued, attempts at resistance offer opportunities to investigate how power operates [24]. In this case, Adam’s position as a genderqueer man provides an opportunity to explore how normative constructions of masculinity perpetuate male violence, as well as how these constructions can be resisted.

## Objective

To explore the relationship between Adam’s genderqueer identity and notions of masculinity and violence, this article analyses an extract from his interview in which he discusses responding to an altercation with another man. The article has two main aims. First, it aims to demonstrate the shifting relationship between constructions of violent and non-violent masculinity in the talk of a genderqueer man, as well as the differing subjectivities contained within these competing discursive constructions. Chris Weedon has defined subjectivity as ‘the conscious and unconscious thoughts and emotions of the individual, (their) sense of (them)self and (their) way of understanding (their) relation to the world’ [25, p. 320]. Secondly, by using a case study, it aims to show how qualitative approaches can have a decisive advantage in that they are able to reveal the complexity and contradiction of masculine identities, as these are negotiated within the context of the interview. The qualitative examination of the complex, shifting relationship between gender equality, masculinities and men’s violence is warranted in order to advance more meaningful violence prevention initiatives.

Research which explores the ways in which violent men talk about violence is significant for two reasons. First, it offers insights into men’s subjective understandings of their use of violence, which tend to be overlooked in quantitative work on men and violence. Secondly, it reveals how men position themselves in relation to their use of violence and how they construct their subjectivities post-violence [26]. Similarly, by focusing on a case of a ‘non-violent man’ in a violent context it becomes possible to examine the social discourses that are embedded in talk, as well as inscribed into subjectivity. By this, we mean the ways in which particular constructions of masculinity ‘categoris[e] the individual, mark[s] him by his own individuality, attaches to him his own identity, imposes a law of truth on him which he must recognise and which others have to recognise in him’ [24, p. 781]. Thus, we are concerned with how power is organised at the level of the individual [27].

## Methods

The case study of Adam emerges from a larger study which explored how gender is constructed by both parents and children within families [28]. In particular, the study investigated how particular constructions of gender promote or prevent gender equality within the context of the home. The study received ethical clearance from the University of South Africa. The study included 18 families from a range of different cultural and material backgrounds in Cape Town. The families included in the study all had children aged between 6 and 17 years. Families who were likely to identify with notions of gender equality were recruited by posting an advert on two social media pages which focus on feminist issues. Adam’s family was one who identified with notions of gender equality and identified themselves as a ‘feminist family’. Other families were recruited through religious and community-based organisations, as well as a snowball sampling technique. It was explained to participants that they would be interviewed about their own and their families’ views and practices of gender and gender (in)equality (for example, how they think and talk about gender, how they divide up tasks at home, how their gender affects how they parent/are parented). All the families that were recruited agreed to be interviewed. Parents signed consent forms for themselves, as well as their children, and children signed assent forms.

Like all other interviews in the study, Adam’s interview was conducted at his home. Adam’s interview lasted for approximately an hour. The interview questions covered a range of topics related to meanings and practices of gender within the family. Parents were asked the following questions: Tell me about how you raise your children; do you think there should be a difference in how boys and girls are raised? How do you think the fact that your child is a boy/girl affects the way you raise him/her? How do you think the fact that you are a woman/man affects the way in which you parent? (If applicable) How do you and your partner differ in your approach to parenting? Children were asked: What do you like about your family? Who is the boss in your family? Can you tell me about your parents? Do you think the fact that you are a boy/girl affects the way in which your parent treat you? Do you think boys and girls should be treated differently? Do you think that you will have children someday? What type of parent would you like to be? Following the interviews, participants were given the option of reading the interview transcript or listening to the interview recording. However, none of the participants requested to read or listen to the interviews.

Adam’s interview has been analysed using a Foucauldian discourse analysis, focusing on how the subject constitutes himself, as well as how this constitution is related to broader structures of power [29]. Adam’s interview was transcribed by the first author and then analysed by both authors according to six steps: identifying discursive constructions; situating these constructions in relation to particular discourses; examining the discursive contexts in which discursive constructions are deployed; identifying the subject positions offered by particular discursive constructions; exploring the way in which discursive constructions create or prevent opportunities for action; and identifying what can be thought, felt and experienced within a particular subject position [30]. The analysis focuses on how Adam responded to questions about his gender identity and how he relates that to violence.

The analysis probes Adam’s description of a particular incident which he sees as demonstrative of ‘a male point of view’. Adam’s description is uniquely interesting as it also reflects on male violence. Simultaneously, the extract also illustrates the dynamic interaction between interviewer and interviewee. This points to how qualitative approaches can have a decisive advantage in that they are able to reveal the complexity and contradiction of masculine identities.

## Results and discussion

Adam’s discussion of the incident presented below was precipitated by a question about how his gender affects the way in which he parents his children. He explained that he does not know how to explain ‘traditional male behaviour’ to his daughters.

Adam: I think there there’s some stuff that that you sort of learn to accept with a male point of view which perhaps my daughters have never… learnt well to understand or accept because you know it’s just so foreign.

Interviewer: They haven’t been exposed to that?

Adam: Ja.

Interviewer: Ja? Can you give me an example with some of the things that you’re sort of talking about?

Adam: Um … well here’s one that I’ve got a bit myself is um male aggression. So the other day we had an altercation with a one of the guys […] walking our dog. He’d chased us down in his car because he said I was training my dog outside his gate and it was setting his dogs off and I mean it was a bit ridiculous everything and everything and um talking to peop- other people who walk their dogs on the field you know my first instinct was to sort of fight back um and talking to other people who walk their dogs on the field, it was starkly gender divided with the men saying that’s ridiculous and you must stand up and fight back and the women saying well what if he has a gun? What if you know something goes wrong and you get badly hurt? Like you don’t know this guy from … [laughs]

Interviewer: Ja the sort of well I guess it’s like the f- the flight or fight sort of response?

Adam: Yes. Yes. Ja. Ja and and I think intrinsic in our culture is this um impression that men … must stand up for their rights and women must be careful about who could attack them … you know the sort of weak–weaker gender stereotype.

Interviewer: Ja. Ja. So you’re saying that even though … um … perhaps in general you don’t sort of identify with sort of traditionally masculine things, that’s still something which on some level you kind of feel like is a sort of response of yours?

Adam: Yes it is a response of mine and I I mean I think I think my response is mostly privileged based because I’ve I haven’t had to be scared. I haven’t been told by others to be scared because I present as male, you know. I haven’t had the experience of people saying well you know what did you think you were doing go- going down that alley or well what did you expect exposing yourself in that sort of way? It’s sort of been more expected that I’d be able to defend myself. If I found myself in a dangerous situation.

### Masculinities and violence

The extract shows the complex ways in which Adam constructs his masculinity. Throughout the interview he grapples with contradictory notions as he reflects on his response to an altercation with another man.

Adam notes that ‘you sort of learn to accept *a male point of view*’. He argues that this point of view is ‘foreign’ to his daughters, positioning it as linked to maleness. Therefore, this male point of view is constructed as static, fixed and inherently connected to men’s bodies. In contrast, Adam also notes that you ‘*learn to accept*’ this point of view. In other words, the male point of view is socially learned. Therefore, while Adam describes the ‘male point of view’ as inflexible, he also positions himself as having some sense of agency in relation to this point of view. Similarly, he explains that he has ‘a bit’ of ‘male aggression’. The use of the term ‘a bit’ constructs ‘male aggression’ as potentially variable and flexible. However, the notion of aggression is anchored to maleness through Adam referring to it as ‘male aggression’. Adam is thus drawing on constructions of aggression as a key component of masculinity. Dominant constructions of masculinity are ‘typically associated with characteristics such as dominance, aggression, assertiveness, and self-assurance’ [31,p.1020]. Therefore, Adam is wrestling with masculinity as inherently predetermined, automatically connected with men and therefore immoveable, while simultaneously constructing it as vacillating and adjustable. This *struggle* has important implications for the way in which men are able to position themselves in relation to violent or non-violent masculinities.

Adam notes that, in response to another man accusing him of training his dog outside his gate, ‘my first *instinct* was to sort of *fight back*’. ‘Instinct’ signals that Adam draws on a biological discourse of male aggression which positions men’s violence as predisposed and rooted in male physiology [32]. Therefore, this violent response is constructed as ‘instinctive’ and the individual man’s responsibility for engaging in this violence is diminished. This construction is consistent with research that has shown that a key obstacle to transforming gender relations is an account of masculinity as fixed and biologically determined [33]. By positioning masculinity as rooted in biology, the responsibility for certain ‘masculine behaviours’ is constructed as beyond the control of the individual. Therefore, this biological discourse of masculinity can be seen to produce and legitimate an aggressive and violent subjectivity. The discourse inhibits more emotionally vulnerable subjectivities and therefore could prevent men from engaging in alternative, non-violent ways of being [27].

However, Adam constructs male violence as shaped by cultural norms. He says ‘I think intrinsic in our *culture* is this um impression that men … must stand up for their rights’. The words ‘impression’ and ‘culture’ establish male violence as a psychosocial phenomenon, rather than a biological fact. Therefore, rather than an uncontrollable instinct, violence is constructed as a choice. Constructing male violence as rooted in particular psychosocial and cultural assumptions, rather than as an automatic biological response, enables Adam to resist this violence. Through references to ‘*our* culture’ and ‘men’ rather than to himself as an individual, Adam appears to be distancing himself from an instinctively violent version of masculinity. He positions himself as an individual within a broader cultural framework that influences gender relations. Therefore, while Adam constructs gender as socially constituted and therefore flexible, he also highlights the way in which the individual is acted upon by the culture system. In this way, the subjective possibility for resisting violence is created. However, here Adam is also suggesting that doing masculinity ‘is not *simply* a matter of choice but involves grappling with both subjective constraints and the constraints of accepted discursive practices’ [34, p. 249] (emphasis added). This is supported by a study with men in South Africa which has shown that the construction of masculinity involves an ongoing negotiation of hegemonic norms and values [27].

Adam notes that he has not ‘had to be scared […] because I *present* as male’. Rather than as something that he ‘is’, his masculinity is projected as a performance [35]. Here, Adam is also highlighting the way in which a particular performance of masculinity affords him power and, to some extent, protection. Research with transgender people has highlighted the importance of passing; being regarded in line with one’s own self-identified gender/sex [36]. Therefore, by being recognised as a man, Adam is able to occupy an unfearful subjectivity in relation to the threat of violence, in a way that women and more visibly gender-non-conforming people are not able to. However, other research has shown that versions of masculinity predicated on bravery, toughness and the defence of honour facilitate risk-taking behaviour, including engaging in physical violence rather than resolving conflict peacefully [3]. In this way, unfearful masculine subjectivities place men at risk for violence.

Adam again points to the psychosocial rather than the biological nature of male violence when he says: ‘[i]t’s sort of been more *expected* that I’d be able to defend myself’. Adam’s genderqueer identity emerges as central to this construction of masculinity. In the space between Adam’s presentation and experience of gender, the fixedness of the category of masculinity is deconstructed. Adam’s case exemplifies how the ‘in-betweenness’ of genderqueer individuals facilitates a critical consciousness with regard to categories of identity [19]. This is an example of how Adam’s contradictory masculinity, and, in particular, his resistance to a violent version of masculinity, makes clearer the functioning of gendered power [24]. This is supported by research with young genderqueer people, which has demonstrated that these identity positions facilitate a ‘cognitive and emotional reorganizing [of] gender identity’ [18, p. 97].

### Relational negotiations of masculinities

While Adam’s construction of a non-violent masculinity is facilitated by his position as a genderqueer man, this particular construction is also enabled by the relational context of the interview. Adam’s construction emerges out of a conversation between him and the interviewer, in which the interviewer also draws on and contests dominant discourses. The interviewer reproduces the physiological discourse of violent masculinity by saying ‘the flight or fight sort of response’. This is met with a rather emphatic agreement from Adam: ‘Yes. Yes. Ja. Ja’. In the process of conversation, interview and interviewer appear to mutually reinforce each other’s notions of violent masculinity. However, after Adam’s enthusiastic affirmative response about cultural gender stereotypes, the interviewer clarifies and says: ‘perhaps in general you don’t sort of identify with sort of traditionally masculine things, that’s still something which on some level you feel like is a sort of response of yours?’. The interviewer opens up a possibility for a ‘non-violent masculinity’, while simultaneously pointing out the operation of a physiological discourse. In this moment, an opportunity is presented for Adam to further explore and negotiate this liminal space between competing versions of masculinity.

It is important to note that the interviewer’s identity shapes the relational negotiation of masculinity within the interview. In this case, the interview was conducted by the first author, a middle-class, white woman in her late twenties. Not only does she share race and class characteristics with Adam, but both before and during the interview she expressed her identification with feminist and gender egalitarian values. These shared identities shape the interview context in significant ways. For example, Adam’s reference to ‘our culture’ may indicate his recognition that he and the interviewer come from the same cultural backgrounds. The shared feminist and gender egalitarian values of the interviewer and interviewee also make it possible for the interviewer to present masculinity as socially constructed, rather than biological and fixed, for example when she refers to ‘traditionally masculine things’. Similarly, it might not have been possible for Adam to speak about his gender as performative (‘I *present* as male’) and, therefore, to challenge a fixed and biological version of masculinity, if he had not felt that this understanding of gender was shared by the interviewer. These are examples of how knowledge is produced in the interaction between the researcher and the participant, and the importance of paying attention to the ways in which the researchers’ ‘values, experiences, interests, beliefs, political commitments, wide aims in life and social identities’ make certain meanings possible within the interview [37,38, p. 10].

A close focus on talk about violence (and non-violence) demonstrates how ‘the things people say and the things people do in all their “messy complexity” enables a deep and rich knowledge’ of masculinities [37, p. 65]. Qualitative studies of masculinity shed light on the processes that constitute masculinities in particular ways, as well as the ways in which men (and women) assign meaning to certain notions of gender [39]. Qualitative approaches are therefore necessary to enable in-depth, rich understandings of complex social realities such as gender and gender inequality [40]. In South Africa, where levels of violence are high, there is a need for more research that provides nuanced interrogations of the densities of men’s violence and the ‘complexities of the social construction of masculinity and often the contradictory experiences of boys and men’ [41, p. 512]. This type of analysis has the potential to disrupt problematic and colonial notions of violent men as exclusively black, uneducated and poor [42]. In South Africa, these racialised and classed discourses have been challenged by recent high-profile cases, such as that of Oscar Pistorius and Patrick Winsani, both of whom were found guilty of murdering their female partners. Oscar Pistorius, or ‘blade runner’ as he is popularly known, is a South African athlete who is particularly famous for being the first amputee runner to compete at an Olympic Games. On 14 February 2013, Pistorius shot and killed his girlfriend, Reva Steenkamp. He was originally found guilty of culpable homicide, but after a state appeal he was convicted of murder and sentenced to 6 years. The case received widespread media attention owing to Pistorius’s status as a South African hero. Patrick Winsani is the former regional leader of the African National Congress Youth League In Johannesburg. Winsani was found guilty in May 2017 of murdering his girlfriend, Nosipho Madleleni. In his judgment, Judge Ismail Mohamed noted that ‘Winsani is educated, but his actions were contrary from what one would expect from an educated person’ [43]. These examples have demonstrated that despite dominant discourses, it is not just certain men who are capable of perpetrating this type of violence.

## Conclusion

The case study of Adam suggests that escaping dominant attitudes towards violent masculinity is difficult – but not impossible. The case study reveals how male violence is intertwined with a particular understanding of masculinity as biological and therefore ‘natural’ and ‘unchangeable’. However, the interview also demonstrates the complex and contradictory process of negotiating and resisting this violent masculinity. This is evidence of how ‘[a]chieving a different version of masculinity, represent[s] a tremendous challenge, an ongoing struggle’ between competing constructions [44, p. 233]. Understanding the complex, contradictory process of negotiation between different versions of masculinity has important implications for effective violence prevention initiatives. In particular, it suggests that there is a need to open up spaces in which men can repeatedly grapple with and reconstruct their masculinities in non-violent ways. There are a number of such programmes which have been implemented in South Africa, including ‘One Man Can’ and ‘Stepping Stones’ [45,46]. However, the programmes have been targeted exclusively at cis-gendered (and heterosexual) men. In this way these programmes, focused on the promotion of gender equality, do not necessarily deconstruct the essentialism present in the dichotomous order [47]. The development of more inclusive programmes, which target a range of gender identities has the potential to advance alternative and new ways of thinking about gender (and violence) [18].

This article has also shown that the interview itself operates as an important site in which masculinities can be reconstructed in non-violent ways. A space for self-reflection and interrogation was facilitated by asking Adam about the meanings and practices of masculinity. Other research with genderqueer individuals has demonstrated the benefit of opening up spaces for dialogue about issues related to gender. For example, the shared dialogues of young genderqueer people constituted within research focus groups offered new ways for these young people to think about gender [18]. However, at the same time, the meanings which are possible are constrained by the specific relational context of the interview. This highlights the way in which the interview has the potential to operate as a site of intervention, facilitating the (co-construction) of non-violent masculinities.

## Paper context

Violence is a significant global health burden. There is substantial research documenting a connection between violence and masculinity. However, research has predominantly focused on cis-gendered men. This paper aims to extend this research by providing a case study of violence and masculinity in a genderqueer man. The paper demonstrates that (re)formulating masculinity in non-violent ways is a complex process, which requires the establishment of spaces in which men continuously deconstruct violent versions of masculinity.
